# Variation in plastid genomes in the gynodioecious species *Silene vulgaris*

**DOI:** 10.1186/s12870-019-2193-0

**Published:** 2019-12-19

**Authors:** Manuela Krüger, Oushadee A. J. Abeyawardana, Miloslav Juříček, Claudia Krüger, Helena Štorchová

**Affiliations:** Plant Reproduction Laboratory, Institute of Experimental Botany v.v.i, Czech Academy of Sciences, Rozvojová 263, 16502 Prague, Czech Republic

## Abstract

**Background:**

Gynodioecious species exist in two sexes – male-sterile females and hermaphrodites. Male sterility in higher plants often results from mitonuclear interaction between the CMS (cytoplasmic male sterility) gene(s) encoded by mitochondrial genome and by nuclear-encoded restorer genes. Mitochondrial and nuclear-encoded transcriptomes in females and hermaphrodites are intensively studied, but little is known about sex-specific gene expression in plastids. We have compared plastid transcriptomes between females and hermaphrodites in two haplotypes of a gynodioecious species *Silene vulgaris* with known CMS candidate genes.

**Results:**

We generated complete plastid genome sequences from five haplotypes *S. vulgaris* including the haplotypes KRA and KOV, for which complete mitochondrial genome sequences were already published. We constructed a phylogenetic tree based on plastid sequences of *S. vulgaris*. Whereas lowland *S. vulgaris* haplotypes including KRA and KOV clustered together, the accessions from high European mountains diverged early in the phylogram. *S. vulgaris* belongs among *Silene* species with slowly evolving plastid genomes, but we still detected 212 substitutions and 112 indels between two accessions of this species. We estimated elevated Ka/Ks in the *ndhF* gene, which may reflect the adaptation of *S. vulgaris* to high altitudes, or relaxed selection. We compared depth of coverage and editing rates between female and hermaphrodite plastid transcriptomes and found no significant differences between the two sexes. We identified 51 unique C to U editing sites in the plastid genomes of *S. vulgaris*, 38 of them in protein coding regions, 2 in introns, and 11 in intergenic regions. The editing site in the *psbZ* gene was edited only in one of two plastid genomes under study.

**Conclusions:**

We revealed no significant differences between the sexes in plastid transcriptomes of two haplotypes of *S. vulgaris*. It suggests that gene expression of plastid genes is not affected by CMS in flower buds of *S. vulgaris*, although both sexes may still differ in plastid gene expression in specific tissues. We revealed the difference between the plastid transcriptomes of two *S. vulgaris* haplotypes in editing rate and in the coverage of several antisense transcripts. Our results document the variation in plastid genomes and transcriptomes in *S. vulgaris*.

## Background

Gynodioecy is a plant breeding system, in which hermaphrodite (H) and female (F) individuals co-occur in the same population. It occurs in about 2% of all angiosperm genera [1]. Male sterility in most (but not in all) gynodioecious plant species is encoded by the interaction of mitochondrial-encoded cytoplasmic male sterility (CMS) genes and by nuclear restorer of fertility (*Rf*) genes [2]. CMS is used in agriculture to produce hybrid seed with high yield and it is therefore extensively studied in crops, e.g., rice [3, 4], sunflower [5], maize [6], or sugar beet [7].

Despite a widespread occurrence of gynodioecy among angiosperms, studies of CMS in wild species remain scarce. *Silene vulgaris* (bladder campion) emerged as a model for the study of gynodioecy and CMS in natural populations more than two decades ago [8]. The genus *Silene* is rich in species with diverse mating systems – hermaphroditism, gynodioecy and dioecy [9, 10]. The mating system affects DNA sequence variation in organellar loci, which is often higher in gynodioecious *Silene* species than in their closely related dioecious or hermaphroditic congeners most likely owing to balancing selection acting on CMS loci and the whole organellar genome [11, 12].

Besides the mode of selection, substitution rate is another essential factor which influences DNA sequence variation in plant organelles. Whereas the substitution rate is generally low in plant organellar genomes, it is highly elevated in some phylogenetic lineages, including the genus *Silene* [13]. Particularly two species - *Silene noctiflora* and *Silene conica* – achieved extreme rates of sequence and structural evolution in mitochondrial and plastid genomes [14, 15].

*Silene vulgaris* has an organellar genome substitution rate above the angiosperm average but is relatively slowly evolving compared to other *Silene* species [16, 17]. However, intraspecific structural rearrangements in the mitochondrial genome of *S. vulgaris* are extreme, involving not only frequent losses and gains of intergenic DNA, but also changes in coding sequences [16, 18].

The accessibility of completely sequenced mitochondrial genomes of *S. vulgaris* and progress in RNA-seq methods enable the construction of comprehensive mitochondrial transcriptomes in this species. Such comparisons of transcriptomes between F and H plants in *S. vulgaris* have revealed candidate CMS genes in their mitochondrial genomes and found differences in RNA editing rates between the different haplotypes which increased intraspecific protein variation [18, 19].

The plastid genome of *S. vulgaris* has been studied to a lesser extent than the mitochondrial genome of this species. The complete sequence of the plastid genome of only a single haplotype has been published [15] and no comprehensive plastid transcriptome analysis of this species is available.

To gain more detailed insight into plastid genome evolution in *S. vulgaris*, we assembled complete plastid sequences from five haplotypes of this species, including two accessions from high mountains adapted to high altitudes and genetically distant from the remaining three haplotypes originating from lowland populations [20]. CMS is a complex phenotype, which is associated with profound changes in the expression of some mitochondrial and many nuclear genes [21–23]. However, plastid transcriptomes are rarely studied in connection with CMS and it is not known, whether the transcription of plastid genes is affected by the male-sterile phenotype in gynodioecious plants. We therefore compared plastid transcriptomes of F and H plants in two of the haplotypes of *S. vulgaris* (KRA and KOV), for which mitochondrial transcriptomes are also available [18, 19]. The CMS candidate genes were identified in both haplotypes, which would make possible to relate a potential impact of CMS on plastid transcriptome to specific CMS candidates. We did not detect any significant distinctions between the sexes but found interesting differences between transcriptomes of the two *S. vulgaris* haplotypes.

## Results

### Complete plastid genomes of *S. vulgaris*

We assembled five complete plastid genomes of *S. vulgaris* from Eurasia – the haplotypes D11, VS1, ZE2, KRA and KOV (Table 1). They ranged in length from 151,463 bp to 151,572 bp and contained a long single copy region (LSC), a short single copy region (SSC), and two inverted repeats (IRs) (Table 2). The boundaries between repeat and single copy regions and gene content were identical to the previously published plastid genome of *S. vulgaris* SD2 [15].

**Table 1:** Collection sites of *Silene vulgaris* accessions analyzed in this study

Haplotype	Region	Location	Latitude	Longitude Altitude (m a. s. l)	Reference
D11	Europe	Austria, the Alps Dachstein	47.456802	13.621986 1790	This study
VS1	Europe	Czech Republic Jeseníky Mts. Vřesová studánka (Heidebrünnel)	50.145429	17.134176 1295	This study
ZE2	Europe	Netherlands, Zandvoort	52.374868	4.534094 1	This study
KOV	Europe	Czech Republic Kováry near Prague	50.185833	14.253783 270	[16]
KRA	Asia	Russia Krasnoyarsk, Siberia	Not known	Not known about 290	[18]

**Table 2:** Summary of six plastid genomes of *S. vulgaris* compared to the plastid genome of *S. latifolia.* The numbers of indels and nucleotide substitutions in the complete plastid genome alignments of the respective accessions with *S. vulgaris* SD2 are given

Plastid Genome	Accession Number	Size (bp)	LSC (bp)	SSC (bp)	IR (Bp)	Indels	Substitutions
SD2	JF715057	151,583	82,258	17,309	26,008	–	–
KOV	MH890612	151,463	82,180	17,303	25,990	35	80
ZE2	MK473866	151,572	82,257	17,339	25,986	33	35
KRA	MH890613	151,486	82,215	17,307	25,982	87	114
D11	MK473868	151,484	82,208	17,350	25,963	92	97
VS1	MK473867	151,511	82,256	17,316	25,969	112	212
*S. latifolia*	JF715055	151,736	82,704	17,220	25,906	397	1880

We identified the positions of simple sequence repeats (SSR), short arrays of tandem repeat units, in the six plastid genomes of *S. vulgaris* under study. We found 871 mononucleotide repeats longer than five nucleotides, but only 46 of them were longer than nine nucleotides (Additional file 1: Data Set 1). In addition, there were 62 dinucleotides, eight trinucleotides, eight tetranucleotides, and only one pentanucleotide. SSRs represent useful markers in population genetic studies owing to their variability. Within-individual variations in the number of mononucleotide units, arisen due to heteroplasmy, was observed in most mononucleotide regions. SSRs with a repeat unit higher than two nucleotides, not affected by heteroplasmy, can therefore be recommended for plastid genotyping in *S. vulgaris*. We found 19 positions of di-, tri-, or tetranucleotides, which varied among the analyzed plastid genomes of *S. vulgaris*.

### Phylogenetic relationships and sequence polymorphism

**Fig. 1:**
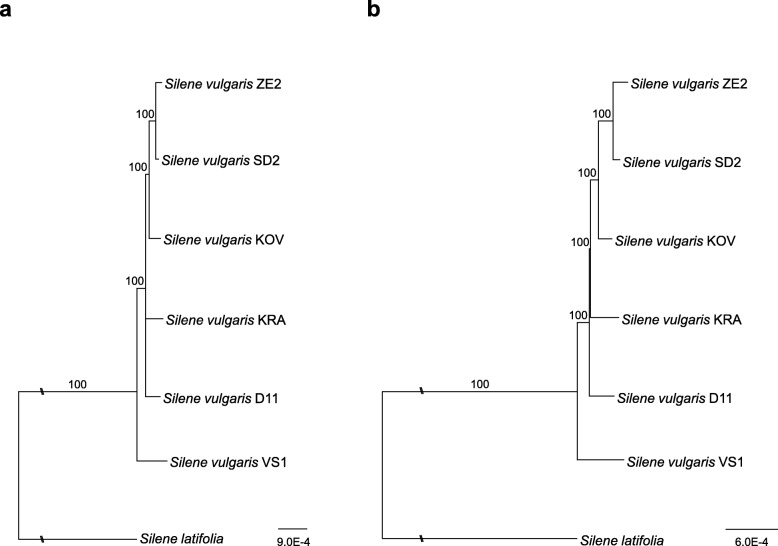
Maximum likelihood phylogenetic trees for various plastid haplotypes of *Silene vulgaris.* **a** based on all sites of the plastid genome except for homopolymer sites larger than five nucleotides; **b** plastid coding regions only. *Silene latifolia* was used as outgroup. Long branches were shortened by 50%, marked by two diagonal slashes. The scale bar indicates the number of substitutions per site. Branches with bootstrap support below 60% were collapsed to polytomies. Phylogenetic trees were computed through the CIPRES webportal with RAxML v. 8.2.10 using 1000 bootstraps and the GTRGAMMA model

Plastid haplotypes VS1 and D11 of the high mountain *S. vulgaris* populations, occurring at the altitudes above 1200 m a. s. l, diverged first on the phylogenetic tree constructed on the basis of concatenated protein-coding sequences and with *S. latifolia* as an outgroup (Fig. 1b). The same topology was confirmed when entire plastid sequences except for homopolymers larger than five were used (Fig. 1a). In the latter phylogenetic tree, the position of the KRA haplotype from Siberia and D11 haplotype from the Alps was not resolved. The results obtained by maximum-likelihood (ML) method were consistent with the outputs generated by MrBayes, except that the position of KRA and D11 was always resolved **(**Additional file 2: Figure S1, Additional file 3: Figure S2).

The number of indels in pairwise alignments of individual plastid genomes of *S. vulgaris* varied from 33 to 112, and nucleotide substitutions ranged from 35 to 212 (Table 2). The highest number of the polymorphisms was found in the alignments with the haplotype VS1 from the Jeseníky Mts, consistent with its divergence’s basal position within phylogenetic tree of *S. vulgaris* haplotypes. The indels occurred in 63 intergenic regions, in 10 introns, and in two coding regions (*ndhD* and *ycf1*), which also showed an elevated number of non-synonymous substitutions (Additional file 1: Data Set 3).

**Fig. 2:**
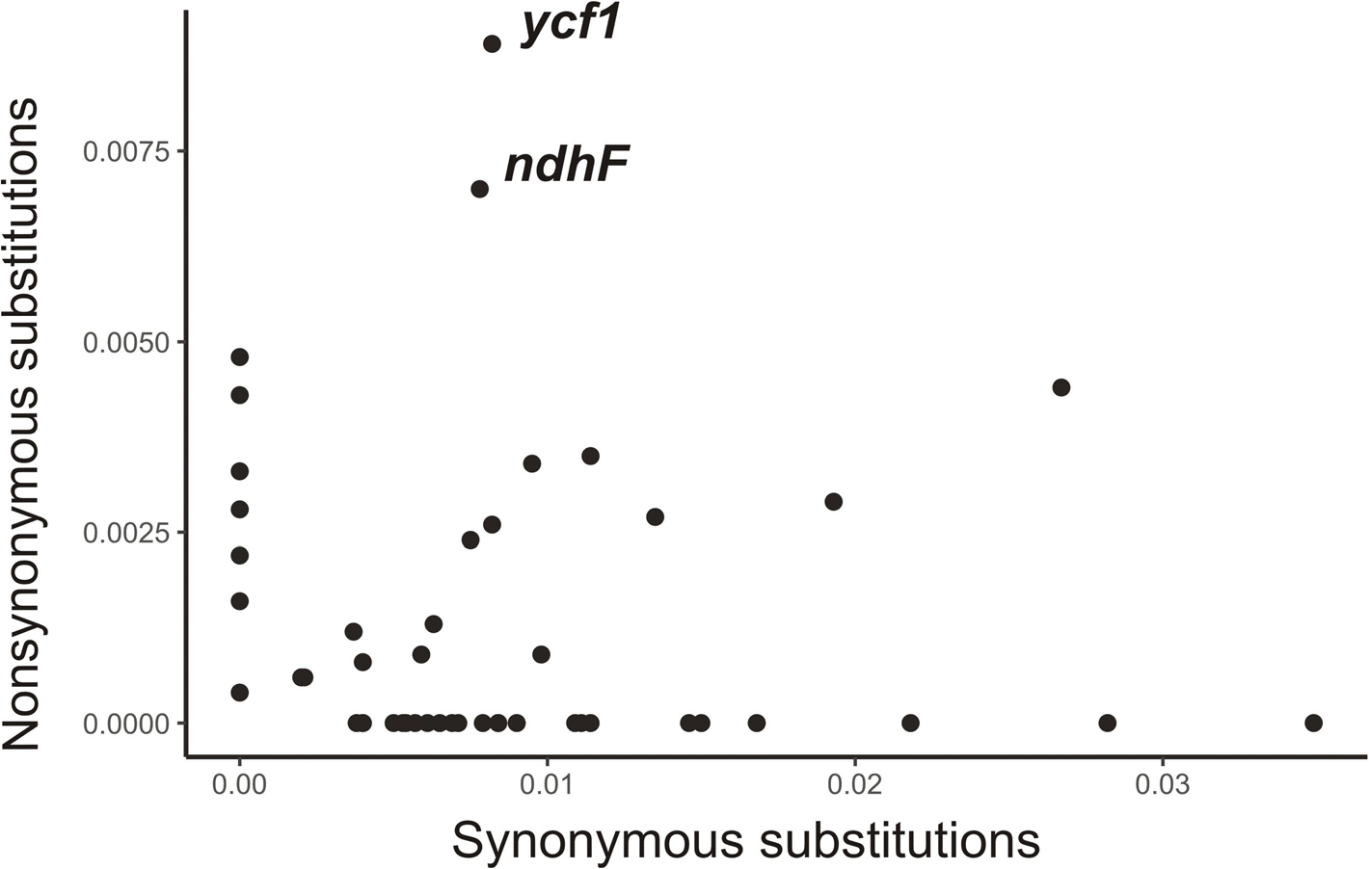
Variation in synonymous and nonsynonymous single nucleotide polymorphisms among *S. vulgaris* plastid protein genes. The number of segregating sites per synonymous or nonsynonymous site was calculated by DnaSp

*S. vulgaris* plastid genes varied in their degree of polymorphism. Thirty of 77 unique protein coding genes were identical, and additional 25 genes carried only synonymous segregating sites and therefore encoded proteins identical among the six plastid haplotypes. Only 22 genes, including *accD*, *matK*, *rpoB* or *ycf2*, carried at least one non-synonymous segregating site (Fig. 2). The substitution in the *rpl20* gene in the haplotypes SD2 and ZE2 created a premature stop codon which shortened the rpl20 protein by the last three amino acids.

Two highly polymorphic genes – *ycf1* and *ndhF* – contained the vast majority of polymorphisms: 48 of the total 69 non-synonymous segregating sites. (Additional file 1. Data Set 2). A detailed inspection of Ka/Ks for these two genes revealed values > 1.0, which indicate relaxed or positive selection. This was the case in all the pairwise comparisons among the *S. vulgaris* haplotypes for *ycf1*. However, only the *ndhF* alignments which comprised the mountain haplotypes VS1 and D11 exhibited high Ka/Ks (Additional file 1: Data Set 4).

### Plastid transcriptomes of *S. vulgaris* KRA and KOV

We generated plastid transcriptomes of two haplotypes of *S. vulgaris* KRA and KOV [18, 19] using the data sets previously employed to construct the mitochondrial transcriptomes. We compared gene coverages and RNA editing rates in flower buds between F and H individuals and between the two haplotypes.

We compared depth of coverage of protein coding genes, because rRNA was removed before cDNA library preparation and small RNAs (< 100 nt) including tRNAs were lost in the course of RNA extraction. Depth of coverage was similar in F and H plants in both haplotype KOV and KRA, no gene was significantly differentially expressed between the sexes. The depth of coverage could not be directly compared between the KOV and KRA plastid genomes, because Illumina sequencing was performed on different platforms and produced reads of different lengths for each plastid transcriptome. We therefore compared the sets of highly and lowly covered genes between the two haplotypes. The genes *psbA*, *rbcL*, *psbE*, *rps14* and *rps16* were among the most highly expressed, whereas the *psbN* was among the least expressed genes both in KRA and KOV plants (Additional file 1: Data Set 5), which indicates general similarity between the two plastid transcriptomes. The *ndhF* gene showed a lower transcript level than other genes encoding the NADH complex proteins as documented by the heat map in Additional file 4: Figure S3.

**Fig. 3:**
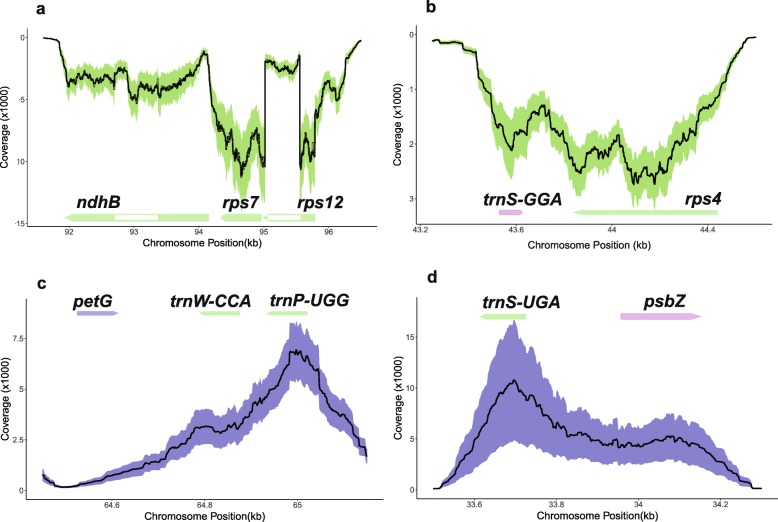
RNA-seq read coverages for genes and antisense transcripts in the plastid genome of *S. vulgaris* KRA. Mean DOC values across individuals are given by the plots’ lines, while standard deviations in DOC are given by the violet (plus strand) or green (minus strand) bands. **a**. The *ndhB*, *rps7*, and *rps12* genes are transcribed from the minus strand. **b**. trnS-GGA is transcribed from the plus strand, but its coverage was not estimated owing to a small size. The antisense *trnS-GGA* transcript has DOC comparable to the adjacent *rps4* gene. **c**. The antisense *trnW-CCA* and *trnP-UGG* co-transcript overlaps the *petG* 3’UTR **d**. The antisense *trnS-UGA* transcript overlaps the *psbZ* 5’UTR

Introns were covered to a lower extent than adjacent exons in most plastid genes, but their coverage reached levels comparable to exons in some genes, for example in *ndhB* (Fig. 3a**).**

### Antisense non-coding transcripts in plastid transcriptomes of *S. vulgaris* KRA and KOV

We identified five antisense non-coding transcripts > 100 nt with transcript abundance comparable to protein coding genes, overlapping *trn* genes (Additional file 1: Data Set 6), three of them were found in both KRA and KOV transcriptomes, two were revealed in KOV only. The *trn*S-GGA and *trn*W-CCA - *trn*P-UGG antisense transcripts corresponded to the 3’UTRs of the *rps4* gene and *petG* genes, respectively. The *trn*S-UGA antisense transcript colocalized with the 5’UTR of the *psbZ* gene (Fig. 3b, c, d**)**.

**Fig. 4:**
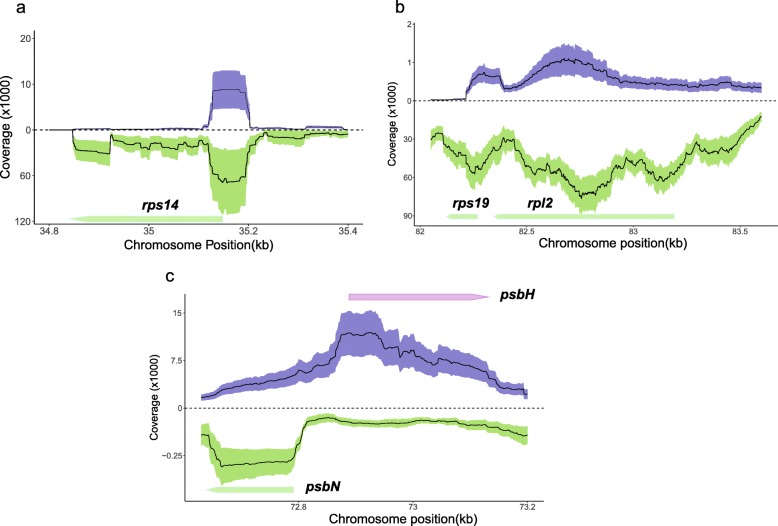
RNA-seq read coverages for genes and antisense transcripts in the plastid genome of *S. vulgaris*. Mean DOC values across individuals are given by the plots’ lines, while standard deviations in DOC are given by the violet (plus strand) or green (minus strand) bands. **a**. The antisense RNA spans the 5’UTR and 5′ end of the *rps14* gene, which is transcribed from the minus strand. **b**. The antisense transcripts of *rps19* and *rpl2* in *S. vulgaris* KRA **c**. The *psbN* gene is transcribed in sense orientation less than in antisense orientation

The antisense transcript spanning the 5’UTR and the start of the *rps14* gene (Fig. 4a**)**, was the most abundant atisense transcript derived from protein coding genes in KOV, but it was absent in the KRA transcriptome. Another antisense transcript derived from the *rps19* and *rpl2* genes was revealed in both *S. vulgaris* transcriptomes under study (Fig. 4b**)**. The *psbN* gene coding for a small transmembrane protein necessary for the assembly of photosystem II [24] was transcribed from minus DNA strand in sense orientation and from the opposite strand in antisense orientation as a part of the longer *psbT*-*psbH* transcript. The antisense *psbN* transcript exhibited much higher depth of coverage than the sense *psbN* transcript coding for the psbN protein (Fig. 4c**).**

Similarly with protein-coding genes, no statistically significant differences in antisense transcript levels were found between F and H plants. In contrast, the abundance of antisense transcripts differed between the KOV and KRA transcriptomes of *S. vulgaris* more than the transcript levels of protein coding genes. The most remarkable distinction was found in the antisense *rps14* transcript (Fig. 4a), which was highly covered in KOV, but completely missing in KRA.

The depth of coverage in the KRA and KOV plastid transcriptome estimated by the ChloroSeq pipeline [25] was in agreement with the results stated above, which were obtained using GSNAP according to [19] (Additional file 1: Data Set 5).

### RNA editing positions in plastid genomes of *S. vulgaris* KRA and KOV

We identified 51 unique C to U editing sites in the plastid genomes of *S. vulgaris* KRA and KOV, 38 of them located in protein coding regions, two of them in introns, and 11 in intergenic regions. Editing sites in rRNAs and tRNAs were not evaluated due to their biased coverage caused by the sample preparation methods. Most edits (95%) in coding sequences were non-synonymous, changing the amino acid composition; only two editing sites were silent. The most frequently edited genes were *ndhB* (9 edits), *ndhD* (4 sites), and *ndhA* (4 sites).

We compared *S. vulgaris* editing sites with eight angiosperm species, for which plastid editome had comprehensively been studied: *Amborella trichopoda*, *Cucumis sativus* [26], *Spirodela polyrhiza* [27], *Aegilops tauschii* [28], *Arabidopsis thaliana* [29], *Hevea brasiliensis* [30], *Nicotiana tabacum* [31], *Vigna radiata* [32] (Table 3). The majority of the 38 edits in protein coding regions identified in *S. vulgaris* were either edited, or C was replaced with T at the DNA level in most angiosperms under comparison. The two silent edits were not conserved across angiosperms. A highly edited position in the *rps16* intron was also edited in *A. tauschii* and replaced with T in DNA of *A. trichopoda* and *A. thaliana*, which may indicate its functional importance. The intergenic regions could not have been reliably aligned across the angiosperm species under comparison.

### *The rate of RNA* editing *in plastid transcriptomes of S. vulgaris KRA and KOV*

The editing rate higher than 80% in at least one of the two *S. vulgaris* transcriptomes was determined in 26 of 38 edits in protein coding genes, all of them were non-synonymous (Additional file 1: Data Set 7). Both silent sites were edited only about 50% or less. An editing event introduced a premature stop codon in about 10% of the *ndhJ* transcripts, but this position was not edited in other angiosperms under comparison. Editing is necessary to create a start codon in the *ndhD* gene in all angiosperms, where C is present in the second position of the coding region. However, all KRA and KOV plants were edited < 15% in this position, which means that only a small portion of the *ndhD* transcripts encoded a functional protein.

In contrast with protein coding genes, only two of 11 editing positions in intergenic regions were edited more than 80%. One of them was located in 3’UTR of the *atpH* gene, the second one in the position 64,933 of the KRA plastid genome in the *trn*W-CCA - *trn*P-UGG antisense transcript.

**Fig. 5:**
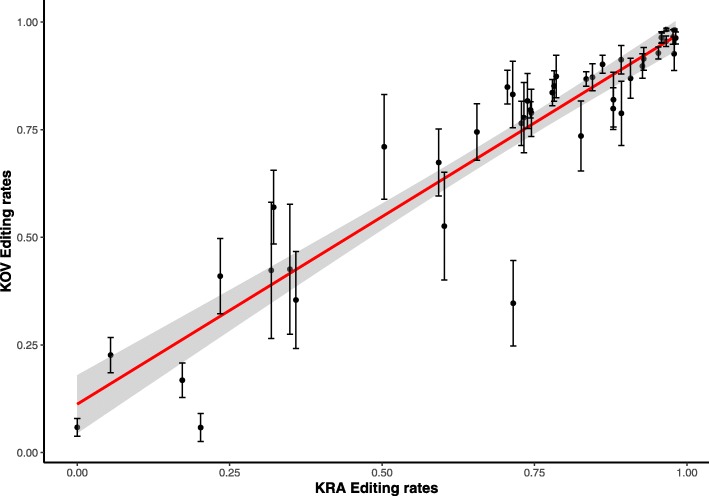
RNA editing extent in *S. vulgaris* plastids. The haplotypes KOV and KRA are compared. Mean values (±SD) calculated from six individuals are plotted, a 95% confidence band for the observed data is given in gray within plot

Editing rates in the KRA and KOV plastid transcriptomes were mutually congruent, exhibiting moderate diffrences in the positions with intermediate rates 40–70% (Fig. 5**)**. The most remarkable difference was found in the position 50 of the *psbZ* coding region, which changed leucine for serine. No editing was observed in this position in the KRA haplotype, whereas approximately 6% of *psbZ* transcripts, which represented about four hundred reads, were edited in each of six KOV plants (Table 3). Editing of this position varied across angiosperms. The same position was edited in *A. thaliana* and *S. polyrhiza*, while no editing was reported in *A. tauschii*, *H. brasiliensis*, or *C. sativus*. T replaced C in this position in the plastid genomes of *N. tabacum* and *V. radiata*. To verify editing of this position in Caryophyllales, we downloaded the transcriptomic data of four *Silene* species, *Agrostemma githago* and *Spinacia oleracea* from the SRA archive and mapped them against the *psbZ* sequence. We found high editing of the position 50 of *psbZ* in *S. conica* and no editing in spinach, *S. noctiflora* and *Silene paradoxa*. This position was edited to a lower extent in *Silene latifolia* and *A. githago* (Additional file 1: Data Set 8). Although the coverage of *psbZ* was low in most data sets, it showed variable editing across close relatives of *S. vulgaris*, the pattern similar to scattered editing at high taxonomic level.

No statistically significant differences of editing rates between F and H individuals were observed between the KOV or KRA haplotypes. The estimates of editing rates provided by the GSNAP [19] and the ChloroSeq pipeline [25] were consistent (Additional file 5: Figure S4).

## Discussion

### Variation in six plastid genomes of *S. vulgaris*

This comparison of six completely sequenced plastid genomes of *S. vulgaris* revealed identical structures and moderate sequence differences, which is in line with the previously reported slow plastid evolutionary rate of this *Silene* species, unlike fast evolving *S. noctiflora* and *S. conica* [15], or *Silene paradoxa* and *Lychnis chalcedonica* [17]. The *S. vulgaris* plastid genome is more similar to the plastid genome of *S. latifolia* in terms of structure and evolutionary rate. Our study involved two accessions from mountain populations and one accession collected in sandy beach close to the sea. The mountain haplotypes diverged first in the phylogenetic tree, confirming their genetic distance from lowland populations. In contrast, the beach accession ZE2 clustered together with the remaining populations, suggesting it recently diverged likely becoming isolated in the process of adaptation to the ecologically divergent seashore habitat with an increased salinity.

High variation in plastid evolutionary rates exist not only among the *Silene* species, but also among individual plastid genes [17]. We found prominent distinctions in substitution rate among the plastid genes also at the within-species level in *S. vulgaris*. Mainly two genes, *ycf1* and *ndhF*, accumulated a high number of non-synonymous substitutions. Whereas the *ycf1* gene exhibited high acceleration in substitution rate across all *Silene* species and also other angiosperms, the *ndhF* gene belonged among the more slowly evolving genes [17]. Our study found elevated Ka/Ks in the *ndhF* pair-wise alignments which included at least one mountain haplotype of *S. vulgaris*. The Ka/Ks values close to 1 indicate relaxed selection, the values > 1 may imply positive selection. Our results suggest distinct selection acting on the *ndhF* gene in mountain populations compared to lowland plants of *S. vulgaris*. The habitats above timberline are exposed to intense light including UV. The increased content of flavonoids in the mountain populations of *S. vulgaris* has been interpreted as a defense against excessive light [33]. It is possible that the non-synonymous substitutions in the *ndhF* may be also related to adaptation to a higher light intensity in high altitudes.

### RNA editing in plastid transcriptomes of *S. vulgaris*

We found no significant difference in the plastid transcriptomes of flower buds in terms of coverage and RNA editing rate between F and H plants neither in the KRA nor KOV haplotype of *S. vulgaris*. This indicates that plastid transcriptomes were not affected by the processes underlying pollen abortion in two haplotypes with distinct CMS types [18, 19]. However, as we analyzed total RNA from entire flower buds, it cannot be excluded that the transcription in plastids of some specific tissues, e.g., tapetum is still influenced by CMS.

Thirty-eight RNA editing sites were revealed in the protein coding genes in the *S. vulgaris* transcriptomes. This value is comparable to those reported in other eudicots: 34 edits in *A. thaliana* [29], 40 edits in *V. radiata* [32], or 51 edits in *C. sativus* [26]. This number is much lower than 138 editing sites discovered in the plastid protein coding genes of the basal angiosperm *A. trichopoda*, which is in agreement with a general trend of gradual loss of plastid edits in the course of the evolution of flowering plants [26]. The loss of editing by the replacement of C for T in genomic DNA occurred in parallel to a similar extent, but it affected distinct sites in various lineages (Table 3).

**Table 4:** Plastid DNA insertions in mitochondrial genomes of *S. vulgaris* KRA and *S. vulgaris* KOV with more than 95% of sequence similarity

*S.vulgaris* KRA			*S. vulgaris* KOV		
Mitochondrial KRA-1 coordinates	Plastid genome coordinates	Size (nt)	Mitochondrial KOV-1 coordinates	Plastid genome coordinates	Size (nt)
100,495 - 100,872	35,346 - 35,723	377	196,739 - 203,697	71,340 - 78,306	6966
295,098 - 295,716	40,335 - 40,982	647	189,999 - 195,890	85,338 - 91,246	5908
368,306 - 371,081	50,900 - 53,664	2764	156,787 - 169,357	93,157 - 105,727	12,570
135,024 - 135,444	73,826 - 74,243	417			
318,767 - 321,559	75,549 - 78,345	2796			
78,752 - 79,770	81,520 - 82,538	1018			

The vast majority of editing sites in protein coding genes (36) were non-synonymous, changing the encoded amino acid. With a single exception (low-rate edits introducing a premature stop codon in *ndhJ*), all non-synonymous sites were conserved - they were either edited or C was replaced with T in at least one species under comparison.

Most, but not all non-synonymous sites were edited more than 80%. An interesting example of a largely unedited essential position was observed in the *ndhD* gene, where editing established the start codon in only about 15% of transcripts, which strongly reduced the abundance of correct mRNA and might have decreased the production of functional NdhD protein. The same position was low-edited in non-photosynthetic organs (roots, etiolated seedlings) of *A. thaliana* [34]. As our transcriptomes were derived from young flower buds, which contained both photosynthetic and non-photosynthetic tissues, low editing of the *ndhD* start codon might have reflected a lack of editing in some floral tissues. Owing to its possible strong impact on the NdhD protein abundance and the function of NDH complex, editing of the start codon of might have been employed to regulate the expression of the *ndhD* gene. Although the primary function of organellar RNA editing in plants resides in the restoration of conserved amino acids [35], its role in posttranscriptional gene expression control shall be considered [36]. Additional examples of developmental stage- and tissue-specific RNA editing were previously described in plastids of tomato [37] or potato [38].

The two haplotypes of *S. vulgaris* differed in editing extent of the *psbZ* gene. One haplotype was edited to a low extent, but consistently across all six individuals of both sexes, the other one was not edited at all. Editing rate of the position 50 of *psbZ* varied across angiosperms under comparison, where all the possibilities, namely editing, replacement of C for T, and zero editing, were observed (Table 4). No comprehensive plastid transcriptome of *Silene* has been published, but mining available transcriptomic data from GenBank showed similar pattern of the variation in *psbZ* editing rate across close relatives of *S. vulgaris* as across angiosperms. The psbZ protein is an important component the supramolecular architecture of photosystem II [39, 40], whose subunits belong among the least divergent genes in *Silene* [17], most likely owing to the action of purifying selection. The variation in *psbZ* RNA editing across angiosperms, Caryophyllaceae, and even within a single species *S. vulgaris* is therefore surprising. The editing of the position 50 replaces S for L in the middle of the first transmembrane domain of psbZ [40]. It is possible that this exchange does not have noticeable impact on the protein function. The variation in RNA editing among *Arabidopsis* species, affecting functionally less important sites, was recently described [41], but the position 50 of *psbZ* was uniformly edited in the *Arabidopsis* species according to this study. As the position 50 of *psbZ* is completely edited in the model plant *A. thaliana*, the identification of nuclear factors responsible for the *psbZ* editing will be possible, which may help to clarify the function of this editing event.

**Table 3:** The comparison of editing between the plastid genomes of *S. vulgaris* KRA and *S. vulgaris* KOV and eight angiosperm species. *Ambo*. – *Amborella trichopoda*, *Spiro*. – *Spirodela polyrhiza*, *Aegilops tauschii*, *Arab.* – *Arabidopsis thaliana*, *Hevea brasiliensis*, *Nicot.* – *Nicotiana tabacum*, *Vigna radiata*, *Cucumis sativus*

		Genome position	Edit rate	Genome position	Edit rate	*Ambo.*	*Spiro.*	*Aegilops*	*Arab.*	*Hevea*	*Nicot.*	*Vigna*	*Cucumis*
Gene position	Effect/Notes	KRA		KOV									
	rps 16 intron	5004	69%	4996	30%	NA	NA	NA	NA	C	NA	NA	NA
	rps 16 intron	5325	93%	5317	93%	T	C	Edit	T	C	C?	C?	C?
atpAeU914SL	TCA (S) = > TTA (L)	10,003	96%	10,000	96%	Edit	T	T	T	T	T	T	T
	intergenic	12,532	92%	12,531	93%	NA	NA	NA	NA	NA	NA	NA	NA
	intergenic	13,336	50%	13,336	72%	NA	NA	NA	NA	NA	NA	NA	NA
rps2eU248SL	TCA (S) = > TTA (L)	14,920	98%	14,920	97%	C	T	T	T	Edit	C	Edit	Edit
rpoC2eU2968HY	CAT (H) = > TAT (Y)	16,608	70%	16,586	85%	C	T	C	T	C	C	C	C
rpoBeU566SL	TCG (S) = > TTG (L)	25,178	52%	25,156	80%	C	T	Edit	T	T	T	Edit	Edit
rpoBeU551SL	TCA (S) = > TTA (L)	25,193	49%	25,171	76%	Edit	T	Edit	Edit	Edit	Edit	Edit	Edit
rpoBeU473SL	TCA (S) = > TTA (L)	25,271	78%	25,249	83%	Edit	Edit	Edit	T	T	Edit	T	T
psbZeU50SL	TCA (S) = > TTA (L)	34,009	0	33,958	6%	C	Edit	C	Edit	C	T	T	C
rps14eU80SL	TCA (S) = > TTA (L)	35,133	98%	35,071	96%	T	T	T	Edit	Edit	Edit	Edit	T
ndhJeU160QX	CAA(Q) = > TAA(Stop)	47,529	13%	47,486	8%	C	C	C	C	C	C	C	C
	intergenic	47,740	32%	47,697	45%	NA	NA	NA	NA	NA	NA	NA	NA
ndhKeU65SL	TCA (S) = > TTA (L)	48,408	65%	48,365	77%	Edit	T	Edit	T	Edit	T	T	Edit
	intergenic	49,285	23%	49,243	43%	NA	NA	NA	NA	NA	NA	NA	NA
accDeU1337PL	CCA (P) = > CTA (L)	57,221	95%	57,185	96%	Edit	T	T	C)^b^	T	T	T	T
psaIeU85HY	CAT (H) = > TAT (Y)	57,642	71%	57,601	82%	Edit	T	T	C	T	C	Edit	Edit
psbLeU2TM	ACG(T) = > ATG (Start)	62,667	96%	62,630	98%	Edit	T	T	T	T	Edit	T	T
petLeU5PL	CCT (P) = > CTT (L)	64,268	86%	64,236	90%	Edit	T	T	Edit	Edit	T	Edit	T
	intergenic	64,933	83%	64,902	86%	NA	NA	NA	NA	NA	NA	NA	NA
clpPeU559HY	CAT (H) = > TAT (Y)	68,351	97%	68,321	98%	Edit	T	T	Edit	Edit	T	Edit	Edit
petBeU12VV	Silent GTC (V) = > GTT (V)	73,958	32%	73,935	56%	A	C	A	T	C	A	T	G
petBeU418RW	CGG (R) = > TGG (W)	74,364	97%	74,341	93%	Edit	T	T	T	T	T	T	Edit
rpoAeU200SF	TCT (S) = > TTT (F)	77,018	93%	76,983	89%	Edit	Edit	T	Edit	Edit	C	Edit	C
ndhBeU1481PL	CCA (P) = > CTA (L)	91,996	89%	91,961	80%	Edit	Edit	Edit	Edit	Edit	Edit	Edit	Edit
ndhBeU836SL	TCA (S) = > TTA (L)	92,641	90%	92,606	88%	T	Edit	Edit	Edit	C	Edit	Edit	C
ndhBeU830SL	TCA (S) = > TTA (L)	92,647	88%	92,612	84%	Edit	Edit	Edit	Edit	Edit	Edit	Edit	Edit
ndhBeU746SF	TCT (S) = > TTT (F)	93,399	74%	93,364	80%	T	T	T	Edit	Edit	Edit	Edit	C
ndhBeU737PL	CCA (P) = > CTA (L)	93,408	87%	93,373	81%	Edit	Edit	Edit	T	T	Edit	Edit	Edit
ndhBeU586HY	CAT (H) = > TAT (Y)	93,559	60%	93,524	56%	Edit	Edit	Edit	Edit	Edit	Edit	Edit	Edit
ndhBeU542TM	ACG (T) = > ATG (M)	93,603	30%	93,568	45%	Edit	Edit	T	T	Edit	T	Edit	Edit
ndhBeU467PL	CCA (P) = > CTA (L)	93,678	73%	93,643	79%	Edit	Edit	Edit	Edit	Edit	Edit	T	Edit
ndhBeU149SL	TCA (S) = > TTA (L)	93,996	58%	93,961	69%	Edit	Edit	Edit	Edit	Edit	Edit	Edit	Edit
ndhDeU1298SL	TCA (S) = > TTA (L)	113,839	96%	113,810	96%	T	T	T	T	T	C	T	T
ndhDeU887PL	CCC (P) = > CTC (L)	114,250	84%	114,221	87%	T	T	T	Edit	Edit	T	T	Edit
ndhDeU383HY	CAT (H) = > TAT (Y)	114,754	78%	114,722	87%	Edit	T	T	Edit	T	Edit	Edit	Edit
ndhDeU2TM	ACG(T) = > ATG (Start)	115,135	13%	115,103	16%	Edit	Edit	T	Edit	T	Edit	Edit	Edit
ndhGeU50TI	ACA (T) = > ATA (I)	116,860	78%	116,828	86%	C	T	T	Edit	T	C)^c^	C)^d^	Edit
ndhAeU1073SF	TCC (S) = > TTC (F)	117,937	83%	117,905	75%	Edit	Edit	Edit	T	T	Edit	Edit	T
ndhAeU961PS	CCT (P) = > TCT (S)	118,049	74%	118,017	83%	Edit	Edit	T	T	Edit	Edit	T	T
ndhAeU566SL	TCA (S) = > TTA (L)	118,444	6%	118,412	21%	Edit	Edit	Edit	T	Edit	T	T	Edit
ndhAeU341SL	TCA (S) = > TTA (L)	119,692	89%	119,660	73%	T	T	T	Edit	C	Edit	Edit	Edit
ndhHeU303II	Silent ATC (I) = > ATT (I)	120,913	29%	120,881	7%	C	C	T	C	C	C	C	C
	Editing below threshold)^a^												
psaIeU82LF	CTT (L) = > TTT (F)	57,639	5%	57,598	4%	C	C	C	C	C	C	C	C
	intergenic	57,712	9%	57,671	15%	NA	NA	NA	NA	NA	NA	NA	NA
	intergenic	57,717	7%	57,676	12%	NA	NA	NA	NA	NA	NA	NA	NA
	intergenic	57,740	4%	57,699	9%	NA	NA	NA	NA	NA	NA	NA	NA
	intergenic	57,745	5%	57,704	9%	NA	NA	NA	NA	NA	NA	NA	NA
	intergenic	57,892	4%	57,854	8%	NA	NA	NA	NA	NA	NA	NA	NA
	intergenic	57,897	2%	57,859	13%	NA	NA	NA	NA	NA	NA	NA	NA

C? information about editing not available

^a^) Editing rate did not achieve the threshold in most plants (using GSNAP or ChloroSeq),

but editing events observed across all 12 individuals of *S. vulgari*s KO and KRA

)^b^ GCA triplet in *Arabidopsis* encoding alanine

)^c^ TCG triplet in *Nicotiana*, encoding serine

)^d^ TCA triplet in *Vigna*, encoding serine

### Antisense RNAs in plastid transcriptomes of *S. vulgaris*

We found the long antisense transcript of the *psbN* gene, which was more abundant than the sense transcript of this gene. The *psbN* gene is located on the strand complementary to the *psbT*-*psbH* intergenic region, which is a part of the conserved *psbB* operon. The transcription of the *psbN* gene in *A. thaliana* is controlled by a specific promoter recognized by the plastid-encoded RNA polymerase together with nucleus-encoded sigma factor SIG3 [42]. The antisense *psbN* transcript was found to affect the cleavage of the *psbT*-*psbH* intercistronic RNA [43] and to influence the translation of *psbT* mRNA in *A. thaliana* [44]. It is therefore possible that antisense *psbN* transcript has a similar regulatory function in *S. vulgaris*. On the contrary, we found only a very low or zero coverage of the strand complementary to the *ndhB* gene. The antisense *ndhB* transcript was observed in *A. thaliana*, tobacco and poplar and may play a role in mRNA stability control [45]. Its expression is influenced by temperature and developmental stage. It may not be expressed in floral buds, or in *S. vulgaris* at all.

We did not estimate the expression of small RNAs including tRNAs, owing to a size limitation, but we detected longer antisense RNAs transcribed from the strand complementary to the *trn* genes. The antisense *trnS-GGA* and antisense *trnW-CCA* are located in 3’UTR of *rps4* and *petG*, respectively. They form secondary structures, which may be recognized by RNA-binding proteins that regulate transcription of plastid mRNAs [46, 47]. Similarly, the antisense *trnS*-*UGA* may stabilize the 5’end of the *psbZ* transcript and influence its translation.

Numerous antisense RNAs were described in plastid transcriptomes, for example 107 putative antisense transcripts in *A. thaliana* [48], or 137 antisense candidates in *Salvia miltiorrhiza* [49]. We detected only eight putative long antisense RNAs in *S. vulgaris*, which might have been caused by two factors. First, we narrowed our search by raising the coverage threshold to the level of protein-coding genes. Second, we carefully eliminated reads derived from plastid inserts in the mitochondrial genome, which can be erroneously recognized as plastid-encoded transcripts.

The accumulation of antisense RNA can be influenced by the environment and developmental stage [44, 45], which may explain, why some antisense RNAs were expressed only in one haplotype of *S. vulgaris*. On the other hand, all the putative antisense RNAs recognized in the *S. vulgaris* transcriptomes were also found in *A. thaliana* [48], which suggests their evolutionary conservation and possible functional importance.

## Conclusions

We found no significant differences between F and H individuals in the plastid transcriptomes prepared from flower buds (where differences between both sexes may be expected) of two haplotypes of gynodioecious plant *S. vulgaris* KRA and KOV, which suggests that CMS was not associated with the changes in plastid gene expression in this species. However, we cannot exclude, that differences in plastid transcriptomes exist in specific tissues of floral buds. We observed differences between the two haplotypes of *S. vulgaris* in the rate of RNA editing of position 50 *psbZ* gene, which is edited in some angiosperms including *A. thaliana*, but not in the others. Differences in the levels of expression of antisense transcripts were also detected among haplotypes. Our results document the variation in plastid transcriptomes at the intraspecific level in *S. vulgaris*.

The plastid haplotypes KRA and KOV, from which the transcriptomes were constructed belong to a main cluster in the phylogenetic tree constructed of complete plastid genome sequences. *S. vulgaris* populations collected in the high European mountains, for which complete plastid genomes were sequenced, were added to phylogenetic analyses to increase intraspecific sampling. They occupy basal positions on the phylogram and may be closer to the ancestor of *S. vulgaris*.

## Methods

### Plant material

We collected seeds of *S. vulgaris* from two populations occurring above a timber line in European mountains (Dachstein and Vřesová studánka, the haplotypes D11 and VS1, respectively), and from one population growing in sand dunes just above sea level in Netherlands (Zandvoort, ZE2) (Table 1). The mountain populations *S. vulgaris* exhibited morphological traits (floral color, leaf shape, procumbent growth) distinct from lowland plants. These populations were sometimes treated as the separate subspecies *S. vulgaris* subsp. *prostrata* or *S. vulgaris* subsp. *glareosa* [50], but unrestricted gene flow and clinal variation in floral color and flavonoid production along altitudinal gradient were documented [20, 33]. We therefore refer to the mountain populations simply as *S. vulgaris* in our study.

Seeds were germinated and cultivated in the greenhouse at the Institute of Experimental Botany (IEB) in Prague, as described previously [19]. *S. vulgaris* collected in Dachstein is procumbent with light violet flowers. It was sometimes classified as *S. vulgaris* subsp. *prostrata* [50]. However, individuals with intermediate phenotypes between higher altitude and lower altitude plants were reported [20], which suggests unlimited gene flow among the populations. The Dachstein plants are very sensitive to moisture, and they grew poorly in the greenhouse. Thus they were cultivated under controlled conditions in IEB cultivation rooms at 21^o^ C, 16/8 h light/ dark, in pots filled with perlite, vermiculite, and coconut coir (1:1:1), fertilized (Kristalon-start and Kristalon-fruit and flower, 1: 1) once per week or every second week. The plant material was determined by Helena Štorchová. Seed of each *S. vulgaris* haplotype are deposited at IEB in Prague and are available upon request. All samples collected and used in this study did not require any special permission. Plant materials used in the current research complied with government regulations.

### Complete plastid genomic sequences from *S. vulgaris* D11, VS1, and ZE2

We performed de novo assembly of three plastid haplotypes of *S. vulgaris*. About 100 mg of young flower buds from a single H individual *S. vulgaris* D11, VS1 or ZE2 (Table 2) were flash frozen in liquid nitrogen and ground with a china pestle and mortar in Lysis buffer (Qiagen Genomic DNA Buffer Set). The protocol for the preparation of high-molecular genomic DNA using Qiagen Genomic Tip (20G) was followed according to the manufacturer’s instructions. About 8 μg of DNA dissolved in 100 μl of 10 μM Tris-HCl buffer (pH = 8.3) was sent to GATC Biotech (Konstanz, Germany) for SMRT sequencing on the Pacific Bioscience RSII P. A similar aliquot of genomic DNA from each of the three accessions of *S. vulgaris* was sent to the Centre of Plant Structural and Functional Genomics IEB in Olomouc for Illumina MiSeq sequencing (2 × 300 cycles, fragment size about 1000 nt) using Nextera chemistry for DNA library preparation.

The SMRT sequencing generated around 39,000 reads with N50 Read length = 16,500 nt for *S. vulgaris* D11; 98,000 reads with N50 Read length = 16,800 nt for *S. vulgaris* VS1; 94,000 reads with N50 Read length = 18,100 nt for *S. vulgaris* ZE2. A hybrid correction pipeline *proovread* [51] was adopted to correct long but error-prone SMRT reads with short but accurate MiSeq reads. The *proovread* output ‘trimmed’ consisting of error corrected reads was used as a local *blast* database and the reads homologous to the *S. vulgaris* plastid genome (JF715057) were identified by *blastn* search with a cutoff e − 20. Canu v 1.3 [52] was applied for the assembly of *proovread* corrected reads. The resulting two contigs corresponded to a long single copy region (LSC) plus inverted repeat (IR), and a short single copy region (SSC). The complete plastid genomic sequences were deposited under the Genbank accession numbers MK473866–8 (ZE2, VS1, D11).

The complete plastid genomic sequence JF715057 [15] derived from *S. vulgaris* carrying the mitochondrial haplotype SD2 [16] served as a reference for the annotation of the newly assembled plastid genomes of *S. vulgaris*.

### Complete plastid genomic sequences from *S. vulgaris* KOV and KRA

The data sets obtained from the Roche 454 GS-FLX platform with Titanium reagents (from constructed 3 kb paired-end libraries) previously used to assemble mitochondrial genomes of the *S. vulgaris* haplotypes KOV [16] and KRA [18] were utilized for the generation of plastid sequences for both haplotypes. Although the DNA specimens were enriched for mitochondrial DNA, they contained plenty of plastid reads, which provided 10–20 × coverage of the plastid genome. Roche’s GS de novo Assembler v.2.6 (‘Newbler’) was used for initial assembly. The resulting contigs were mapped against the available chloroplast genome of *S. vulgaris* (JF715057) and gaps were filled by individual trimmed 454 reads mapping against the same reference. The KOV and KRA plastid genomic sequences were confirmed by re-mapping of the 454 reads against them. Alignments were manually checked for potential SNPs, indels and insertions and edited respectively. Within-individual variation in A or T homopolymers > 5 was often observed, which might reflect possible heteroplasmy, or the co-existence of two or more variant sequences in the same individual. The resulting KOV and KRA complete plastid sequences (GenBank accession numbers MH890612 and MH890613) were used as the reference genomes for the following transcriptomic analyses.

### Chloroplast genome features

The distance matrix with the pairwise comparison of single nucleotide polymorphisms across all different plastid genomes was calculated using snp-dists (v. 0.6, github.com/tseemann/snp-dists), while for the pairwise comparison of indels the dist.dna function of the ape package within R was employed with the “indel” and “indelblock” model. The positions of simple sequence repeats (SSR) were estimated using the microsatellite identification software tool MISA-web [53] with thresholds of the repeat sequence length longer than five nucleotides for mononucleotides, four repeat units for dimer and trinucleotide SSRs, and three repeat units for tetra-, penta- and hexanucleotide SSRs. The genes and coding regions were annotated according to the plastid genome of *S. vulgaris* (JF715057) and validated by the package ReFernment [54], which confirmed the presence of editing sites in start and stop codons.

### Phylogenetic analyses

First, the six plastid genomes of *S. vulgaris* together with *Silene latifolia* as outgroup (JF715055) were aligned with MAFFT v.7.388 [55] using the L-INS-i mode with misaligned sites manually edited. The inverted repeat region A (IRA) region was cut from the resulting nucleotide alignment. For the calculation of phylogenetic trees two different alignments were compared; first with non-informative sites such as homopolymer regions masked when longer than five nucleotides in non-coding sequence (CDS); second with CDS only (without tRNAs and rRNAs). Additionally, indel characters were coded using the “simple indel coding” algorithm as described in [56] for both alignments with 2matrix [57]. The phylogenetic trees were calculated using RAxML [58] and MrBayes [59] at the CIPRES portal [60].

The maximum likelihood (ML) method was applied using the CIPRES webportal with RAxML v. 8.2.10 with 1000 bootstraps and the GTRGAMMA model for both bootstrapping and tree inference. Indels were given in a partition file as binary characters describing indel size and distribution throughout the respective sequence alignment. Alternatively, the Bayesian approach for phylogenetic tree construction was employed through MrBayes v. 3.2.6 [59] using the Markov chain Monte Carlo algorithm and the default model 4by4 for 5000 generations in two runs with trees sampled every 1000 generations. The different partitioned nexus file consisted of the sequence alignment and the indel coding, each. Stationary character frequency was fixed for the indel data set and dirichlet (1.0, 1.0, 1.0, 1.0) and the first 25% of topologies were discarded (burnt in). The analysis was stopped when the standard deviation of split frequencies between the runs was lower than 0.01.

The numbers of synonymous and nonsynonymous substitutions within *S. vulgaris* plastid protein coding genes were determined with DnaSP v5 [61]. The segregating site was identified, if an alternative nucleotide was found in the respective position in at least one of six aligned plastid sequences of *S. vulgaris*.

### Illumina read mapping

We used the reads stored under the Short Read Archive accession number PRJNA321915. They were obtained by Illumina sequencing of cDNA derived from total RNA extracted from flower buds of three F and three H individuals of *S. vulgaris* KRA [18] (GenBank accession numbers SRX3102769 – SRX3102774) and from flower buds of three F and three H individuals of *S. vulgaris* KOV [19] (GenBank accession numbers SRX272140 – SRX272145).

The initial alignment was performed with the assembler GSNAP v. 2017-05-03 [62] in paired-end mode with known splice sites [14]. The plastid genome sequences of the haplotypes KOV and KRA of *S. vulgaris* were used as the references. IRA within the reference plastid genome was cut away to ensure proper read mapping with GSNAP. The resulting alignments were separated by strand using the view function in SAMtools v. 1.9 [63] by filtering according to read-pair orientation utilizing SAM flags as described in [18] These alignments were filtered for potential mitochondrial reads derived from plastid DNA inserted to the mitochondrial genome of the corresponding haplotype (GenBank accession numbers JQ771300 and MH455602) (Table 4). At these known regions all reads not matching to the reference sequence and not presenting potential RNA editing were filtered deploying SAMtools view function, a custom AWK script and seqtk v. 1.2 (https://github.com/lh3/seqtk) for subsequent analyses. The mapped reads were visualized by means of the Integrative Genomic Viewer (IGV) [64].

For comparison we also used the ChloroSeq pipeline [25] to analyze the plastid transcriptomes of *S. vulgaris* KOV and KRA. This pipeline relies on several different, open-source bioinformatic programs, such as SAMtools and BEDtools v. 2.25.0 to run properly. The same reference, known splice sites and filtered reads as for the final GSNAP alignments were used with bowtie v. 2.2.6 [65] and tophat v. 2.1.1 [66] for read mapping as described in [25].

### RNA editing rates

Initial variant discovery was performed with HaplotypeCaller in GenomeAnalysisTK v. 3.7 [67] on the minus- and plus-stranded alignments of *S. vulgaris* KOV and KRA with the minimum call and emit threshold (stand_call_conf) set to 20. All variant sites with C-to-T for the plus- and G-to-A alteration in the minus-strand were manually checked and verified for subsequent final calling of variants with SAMtools v. 1.2 mpileup using the DPR output tag (discontinued since v. 1.3) for the number of high-quality bases per observed allele. In cases of low read mapping, but definite RNA editing the DP4 values were used to evaluate editing rates across all individuals. RNA editing rates were calculated based on these values as counts of Ts divided by the sum of Cs and Ts at the specific editing sites. The final editing sites were used as list for calculation of editing rates with ChloroSeq. Editing rates below a threshold of 5 % (or 10 edited nucleotides, whatever is smaller), or low coverage (less than 200 reads mapped) are not shown in the final results.

### Transcript abundance estimation

The estimation of transcript abundance was done as described in [19] using the coverage function within bedtools and a custom AWK script. The coverage was calculated per-base, averaged over the length of the respective feature of interest, first regardless of strand followed by strand-specific calculations for each sample and normalized as TPM as described in [68]. The average and standard deviation of TPM were calculated for both haplotypes and sexes. Antisense transcripts were recognized if their depth of coverage exceeded 300–500, which corresponded to the TPM values of the least expressed protein coding genes. The web tool Morpheus (https://software.broadinstitute.org/morpheus) was used for heat map construction.

## Supplementary information

**Additional file 1. **Numerical tables. **Data Set S1**. Simple sequence repeats in plastid genomes of *S. vulgaris*. **Data Set S2.** Substitution rates in plastid genes of *S. vulgaris*. **Data Set S3.** KaKs matrices. **Data Set S4.** Gene coverage of plastid genes of *S. vulgaris*. **Data Set S5.** Antisense transcript coverage in plastid genomes of *S. vulgaris*. **Data Set S6.** Editing rates in plastid genomes of *S. vulgaris*. **Data Set S7.** *psbZ* editing extent

**Additional file 2: Figure S1.** Maximum likelihood phylogenetic trees for different plastid haplotypes of *Silene vulgaris.* **a.** based on all sites of the plastid genome except for homopolymer sites larger than five nucleotides; **b.** plastid coding regions only. *Silene latifolia* was used as outgroup. Long branches were shorten by 50%, indicated with two diagonal slashes. Indels were coded after Simmon & Ochoterena (2000). The scale bar indicates the number of substitutions per site. Branches with bootstrap support below 60% were collapsed to polytomies. Phylogenetic trees were computed through the CIPRES webportal with RAxML v. 8.2.10 using 1000 bootstraps and the GTRGAMMA model

**Additional file 3: Figure S2.** Bayesian 50% majority rule phylogenetic trees for different plastid haplotypes of *Silene vulgaris* based on **a.** all sites of the plastid genome except homopolymer regions larger than five nucleotides; **b.** plastid coding regions only; **c.** all sites of the plastid genome except homopolymer regions larger than five nucleotides with simple indel coding after Simmon & Ochoterena (2000); **d.** plastid coding regions only with simple indel coding. *Silene latifolia* was used as outgroup. Long branches were shorten by 50%, indicated with two diagonal slashes. The scale bar indicates the number of substitutions per site. Phylogenetic trees were computed through the CIPRES webportal with MrBayes v. 3.2.6 using 5000 generations

**Additional file 4: Figure S3** Heat maps showing the transcript levels of the plastid *ndh* genes across six individuals of *S. vulgaris* KRA and KOV. The *ndhF* gene is the least expressed gene in both haplotypes, the expression of the other genes varies between the two haplotypes

**Additional file 5: Figure S4.** The comparison of editing rates estimated by the GSNAP and the ChloroSeq pipeline. Mean values (±SD) calculated from six individuals are plotted, a 95% confidence band for the observed data is given in gray within plot

## Data Availability

The data of this study data have been deposited in the NCBI with BioProject accession number PRJNA321915. The RNA-seq reads from hermaphrodites of *S. vulgaris* KRA are stored under the number SRS2438489, the reads from females under the number SRS2438490. The RNA-seq reads from six individuals of *S. vulgaris* KRA are deposited under GenBank accession numbers SRX272140 – SRX272145. The complete plastid genomes can be found under GenBank accession numbers MH890612 and MH890613 (KOV and KRA), and MK473866–8 (ZE2, VS1,D11).

## Abbreviations

*accD*: *acetyl-CoA carboxylase subunit D*
bp: base pair
cDNA: complementary DNA
CMS: Cytoplasmic male sterility
F: Female
H: hermaphrodite
kb: kilobase
LSC: Long single copy
*matK*: *maturase K*
*ndhA*: *NADH dehydrogenase A*
*ndhB*: *NADH dehydrogenase B*
*ndhD*: *NADH dehydrogenase D*
*ndhF*: *NADH dehydrogenase F*
*psbA*: *photosystem II protein A*
*psbE*: *photosystem II protein E*
*psbH*: *photosystem II protein H*
*psbN*: *photosystem II protein N*
*psbT*: *photosystem II protein T*
*psbZ*: *photosystem II protein Z*
*rbcL*: *rubisco subunit L*
*rpoB*: *RNA polymerase B*
*rps14*: *ribosomal protein S14*
*rps16*: *ribosomal protein S16*
rRNA: ribosomal RNA
SSC: Short single copy
SSR: Simple sequence repeat
tRNA: transfer RNA
UTR: Untranslated region

## References

[1] Renner SS (2014). The relative and absolute frequencies of angiosperm sexual systems: Dioecy, monoecy, gynodioecy, and an updated online database. Am J Bot.

[2] Hanson MR, Bentolila S (2004). Interactions of mitochondrial and nuclear genes that affect male gametophyte development. Plant Cell.

[3] Kazama T (2008). Nakamura, Watanabe, M. Sugita KT. Suppression mechanism of mitochondrial ORF79 accumulation by Rf1 protein in BT-type cytoplasmic male sterile rice. Plant J.

[4] Wang K, Gao F, Ji Y, Liu Y, Dan Z, Yang P (2013). ORFH79 impairs mitochondrial function via interaction with a subunit of electron transport chain complex III in Honglian cytoplasmic male sterile rice. New Phytol.

[5] Sabar M, Gagliardi D, Balk J, Leaver C (2003). ORFB is a subunit of F1FO-ATP synthase: insight into the basis of cytoplasmic male sterility in sunflower. EMBO Rep.

[6] Allen JO, Fauron CM, Minx P, Roark L, Oddiraju S, Guan NL (2007). Comparisons among two fertile and three male-sterile mitochondrial genomes of maize. Genetics.

[7] Darracq A, Varré JS, Maréchal-Drouard L, Courseaux A, Castric V, Saumitou-Laprade P (2011). Structural and content diversity of mitochondrial genome in beet: a comparative genomic analysis. Genome Biol Evol.

[8] Charlesworth D, Laporte V (1998). The male-sterility polymorphism of *Silene vulgaris*: analysis of genetic data: from two populations and comparison with *Thymus vulgaris*. Genetics.

[9] Desfeux C, Maurice S, Henry JP, Lejeune B, Gouyon PH (1996). Reproductive Systems in the Genus *Silene*. Evolution of reproductive systems in the genus *Silene*. Proc R Soc B Biol Sci.

[10] Casimiro-Soriguer I, Buide ML, Narbona E. Diversity of sexual systems within different lineages of the genus *Silene*. AOB Plants. 2015;7:plv037. doi:10.1093/aobpla/plv037 10.1093/aobpla/plv037 PMC4433491 25862920

[11] Städler T, Delph LF (2002). Ancient mitochondrial haplotypes and evidence for intragenic recombination in a gynodioecious plant. Proc Natl Acad Sci U S A.

[12] Touzet P, Delph LF (2009). The effect of breeding system on polymorphism in mitochondrial genes of silene. Genetics..

[13] Mower JP, Touzet P, Gummow JS, Delph LF, Palmer JD (2007). Extensive variation in synonymous substitution rates in mitochondrial genes of seed plants. BMC Evol Biol.

[14] Sloan DB, Alverson AJ, Chuckalovcak JP, Wu M, McCauley DE, Palmer JD, et al. Rapid evolution of enormous, multichromosomal genomes in flowering plant mitochondria with exceptionally high mutation rates. PLoS Biol. 2012;10: e1001241. doi:0.1371/journal.pbio.1001241. 10.1371/journal.pbio.1001241 PMC3260318 22272183

[15] Sloan DB, Alverson AJ, Wu M, Palmer JD, Taylor DR (2012). Recent acceleration of plastid sequence and structural evolution coincides with extreme mitochondrial divergence in the angiosperm genus *Silene*. Genome Biol Evol..

[16] Sloan DB, Müller K, McCauley DE, Taylor DR, Storchová H (2012). Intraspecific variation in mitochondrial genome sequence, structure, and gene content in *Silene vulgaris*, an angiosperm with pervasive cytoplasmic male sterility. New Phytol.

[17] Sloan DB, Triant DA, Forrester NJ, Bergner LM, Wu M, Taylor DR (2014). A recurring syndrome of accelerated plastid genome evolution in the angiosperm tribe Sileneae (Caryophyllaceae). Mol Phylogenet Evol.

[18] Štorchová H, Stone JD, Sloan DB, Abeyawardana OAJ, Muller K, Walterová J, Pažoutová M (2018). Homologous recombination changes the context of cytochrome b transcription in the mitochondrial genome of *Silene vulgaris* KRA. BMC Genomics.

[19] Stone JD, Koloušková P, Sloan DB, Štorchová H (2017). Non-coding RNA may be associated with cytoplasmic male sterility in *Silene vulgaris*. J Exp Bot.

[20] Abbate JL, Antonovics J (2014). Elevational disease distribution in a natural plant–pathogen system: insights from changes across host populations and climate. Oikos.

[21] Li ZF, Zhang YC, Chen YQ (2015). MiRNAs and lncRNAs in reproductive development. Plant Sci.

[22] Wu J, Zhang M, Zhang B, Zhang X, Guo L, Qi T (2017). Genome-wide comparative transcriptome analysis of CMS-D2 and its maintainer and restorer lines in upland cotton. BMC Genomics.

[23] Hamid R, Tomar RS, Marashi H, Malekzadeh S, Golakiya BA, Mohsenpour M (2018). Transcriptome profiling and cataloging differential gene expression in floral buds of fertile and sterile lines of cotton (*Gossypium hirsutum* L.). Gene.

[24] Plöchinger M, Schwenkert S, von Sydow L, Schroder WP, Meurer J (2016). Functional update of the auxiliary TerC and ALB3 in maintenance and assembly of PSII. Front Plant Sci.

[25] Castandet B, Hotto AM, Strickler SR, Stern DB (2016). ChloroSeq, an optimized chloroplast RNA-Seq bioinformatic pipeline, reveals Remodeling of the organellar transcriptome under heat stress. G3-Genes Genomes Genet.

[26] Hein A, Polsakiewicz M, Knoop V (2016). Frequent chloroplast RNA editing in early-branching flowering plants: pilot studies on angiosperm-wide coexistence of editing sites and their nuclear specificity factors. BMC Evol Biol.

[27] Wang W, Zhang W, Wu Y, Maliga P, Messing J (2015). RNA editing in chloroplasts of *Spirodela polyrhiza*, an aquatic monocotelydonous species. PLoS One.

[28] Wang M, Liu H, Ge L, Xing G, Wang M, Weining S (2017). Identification and analysis of RNA editing sites in the chloroplast transcripts of *Aegilops tauschii* L. Genes.

[29] Ruwe H, Castandet B, Schmitz-Linneweber C, Stern DB (2013). Arabidopsis chloroplast quantitative editotype. FEBS Lett.

[30] Tangphatsornruang S, Uthaipaisanwong P, Sangsrakru D, Chanprasert J, Yoocha T, Jomchai N (2011). Characterization of the complete chloroplast genome of *Hevea brasiliensis* reveals genome rearrangement, RNA editing sites and phylogenetic relationships. Gene..

[31] Hirose T, Kusumegi T, Tsudzuki T, Sugiura M (1999). RNA editing sites in tobacco chloroplast transcripts : editing as a possible regulator of chloroplast RNA polymerase activity. Mol Gen Genet.

[32] Lin C, Ko C, Kuo C, Liu M, Schafleitner R (2015). Transcriptional slippage and RNA editing increase the diversity of transcripts in chloroplasts : insight from deep sequencing of *Vigna radiata* genome and transcriptome. PLoS One.

[33] Berardi AE, Fields PD, Abbate JL, Taylor DR (2016). Elevational divergence and clinal variation in floral color and leaf chemistry in *Silene vulgaris*. Am J Bot.

[34] Tseng CC, Lee CJ, Chung YT, Sung TY, Hsieh MH (2013). Differential regulation of Arabidopsis plastid gene expression and RNA editing in non-photosynthetic tissues. Plant Mol Biol.

[35] Maier RM, Neckermann K, Igloi GL, Kossel H (1995). Complete sequence of the maize chloroplast genome: gene content, hotspots of divergence and fine tuning of genetic information by transcript editing. J Mol Biol.

[36] Sloan DB (2017). Nuclear and mitochondrial RNA editing systems have opposite effects on protein diversity. Biol Lett.

[37] Kahlau S, Bock R (2008). Plastid transcriptomics and translatomics of tomato fruit development and chloroplast-to-chromoplast differentiation : chromoplast gene expression largely serves the production of a single protein. Plant Cell.

[38] Valkov VT, Scotti N, Kahlau S, Maclean D, Grillo S, Gray JC (2009). Genome-wide analysis of plastid gene expression in potato leaf chloroplasts and tuber amyloplasts: transcriptional and posttranscriptional control. Plant Physiol.

[39] Swiatek M, Kuras R, Sokolenko A, Higgs D, Olive J, Cinque G (2001). The chloroplast gene *ycf9* encodes a photosystem II ( PSII ) core subunit, PsbZ, that participates in PSII supramolecular architecture. Plant Cell.

[40] Wei X, Su X, Cao P, Liu X, Chang W, Li M (2016). Structure of spinach photosystem II – LHCII supercomplex at 3.2 Å resolution. Nature.

[41] Kawabe A, Furihata HY, Tsujino Y, Kawanabe T, Fujii S, Yoshida T (2019). Divergence of RNA editing among *Arabidopsis* species. Plant Sci.

[42] Zghidi W, Merendino L, Cottet A, Mache R, Lerbs-Mache S (2007). Nucleus-encoded plastid sigma factor SIG3 transcribes specifically the *psbN* gene in plastids. Nucleic Acids Res.

[43] Chevalier F, Ghulam MM, Rondet D, Pfannschmidt T, Merendino L, Lerbs-Mache S (2015). Characterization of the *psbH* precursor RNAs reveals a precise endoribonuclease cleavage site in the *psbT/ psbH* intergenic region that is dependent on *psbN* gene expression. Plant Mol Biol.

[44] Zghidi-Abouzid O, Merendino L, Buhr F, Ghulam MM, Lerbs-Mache S (2011). Characterization of plastid *psbT* sense and antisense RNAs. Nucleic Acids Res.

[45] Georg J, Honsel A, Rennenberg H, Hess WR (2010). Rapid report a long antisense RNA in plant chloroplasts. New Phytol.

[46] Bollenbach TJ, Sharwood RE, Gutierrez R, Lerbs-Mache S, Stern DB (2009). The RNA-binding proteins CSP41a and CSP41b may regulate transcription and translation of chloroplast-encoded RNAs in Arabidopsis. Plant Mol Biol.

[47] Manavski N, Schmid LM, Meurer J (2018). RNA-stabilization factors in chloroplasts of vascular plants. Essays Biochem.

[48] Hotto AM, Schmitz RJ, Fei Z, Ecker JR, Stern DB (2011). Unexpected diversity of chloroplast noncoding RNAs as revealed by deep sequencing of the *Arabidopsis* transcriptome. G3-Genes Genomes Genet.

[49] Chen H, Zhang J, Yuan G, Liu C (2014). Complex interplay among DNA modification, noncoding RNA expression and protein-coding RNA expression in *Salvia miltiorrhiza* chloroplast genome. PLoS One.

[50] Marsden-Jones EM, Turrill WB (1957). The bladder campions.

[51] Hackl T, Hedrich R, Schultz J, Forster F (2014). Sequence analysis proovread : large-scale high-accuracy PacBio correction through iterative short read consensus. Bioinformatics.

[52] Koren S, Walenz BP, Berlin K, Miller JR, Bergman NH, Phillippy AM (2017). Canu: scalable and accurate long-read assembly via adaptive k -mer weighting and repeat separation. Genome Res.

[53] Beier S, Thiel T, Munich T, Scholz U, Mascher M. Sequence analysis MISA-web: a web server for microsatellite prediction. Bioiformatics. 2017;33:2583–2585. doi: 0.1093/bioinformatics/btx198. 10.1093/bioinformatics/btx198 PMC5870701 28398459

[54] Robison TA, Wolf PG (2019). ReFernment: an R package for annotating RNA editing in plastid genomes. Appl Plant Sci.

[55] Katoh K, Standley DM (2013). MAFFT multiple sequence alignment software version 7: improvements in performance and usability article fast track. Mol Biol Evol.

[56] Simmons MP, Ochoterena H (2000). Society of Systematic Biologists gaps as characters in sequence-based phylogenetic analyses. Syst Biol.

[57] Salinas DR, Little DP (2014). 2MATRIX : A utility for indel coding and phylogenetic MATRIX concatenation. Appl Plant Sci.

[58] Stamatakis A (2014). RAxML version 8 : a tool for phylogenetic analysis and post-analysis of large phylogenies. Bioinformatics.

[59] Ronquist F, Teslenko M, van der Mark P, Ayres DL, Darling A, Hohna S (2012). MrBayes 3.2: efficient bayesian phylogenetic inference and model choice across a large model space. Syst Biol.

[60] Miller MA, Pfeiffer W, Schwartz T. Creating the CIPRES Science Gateway for inference of large phylogenetic trees. Proceedings of the Gateway Computing Environments Workshop (GCE), 14 Nov. 2010, New Orleans. LA: IEEE; p 1–8. 10.1109/GCE.2010.5676129.

[61] Librado P, Rozas J (2009). DnaSP v5: a software for comprehensive analysis of DNA polymorphism data. Bioinformatics..

[62] Wu TD, Nacu S (2010). Fast and SNP-tolerant detection of complex variants and splicing in short reads. Bioinformatics..

[63] Li H, Handsaker B, Wysoker A, Fennell T, Ruan J, Homer N (2009). The sequence alignment / map format and SAMtools. Bioinformatics.

[64] Thorvaldsdottir P (2012). Integrative genomics viewer (IGV): high-performance genomics data visualization and exploration. Brief Bioinform.

[65] Langmead B, Salzberg SL (2012). Fast gapped-read alignment with bowtie 2. Nat Methods.

[66] Trapnell C, Pachter L, Salzberg SL (2009). TopHat : discovering splice junctions with RNA-Seq. Bioinformatics..

[67] McKenna A, Hanna M, Banks E, Sivachenko A, Cibulskis A, Kernytsky A (2010). The genome analysis toolkit: a MapReduce framework for analyzing next-generation DNA sequencing data. Genome Res.

[68] Wagner GP, Kin K, Lynch VJ (2012). Measurement of mRNA abundance using RNA-seq data: RPKM measure is inconsistent among samples. Theory Biosci.

