# Supplementary material for: Variation in plastid genomes in the gynodioecious species Silene vulgaris
Source: BMC Plant Biol. 2019 Dec 19;19:568. doi: 10.1186/s12870-019-2193-0 (PMC6921581; doi:10.1186/s12870-019-2193-0)
Supplement: Supplementary file 2 — Additional file 2: Figure S1. Maximum likelihood phylogenetic trees for different plastid haplotypes of Silene vulgaris. a. based on all sites of the plastid genome except for homopolymer sites larger than five nucleotides; b. plastid coding regions only. Silene latifolia was used as outgroup. Long branches were shorten by 50%, indicated with two diagonal slashes. Indels were coded after Simmon & Ochoterena (2000). The scale bar indicates the number of substitutions per site. Branches with bootstrap support below 60% were collapsed to polytomies. Phylogenetic trees were computed through the CIPRES webportal with RAxML v. 8.2.10 using 1000 bootstraps and the GTRGAMMA model [file 12870_2019_2193_MOESM2_ESM.pdf]

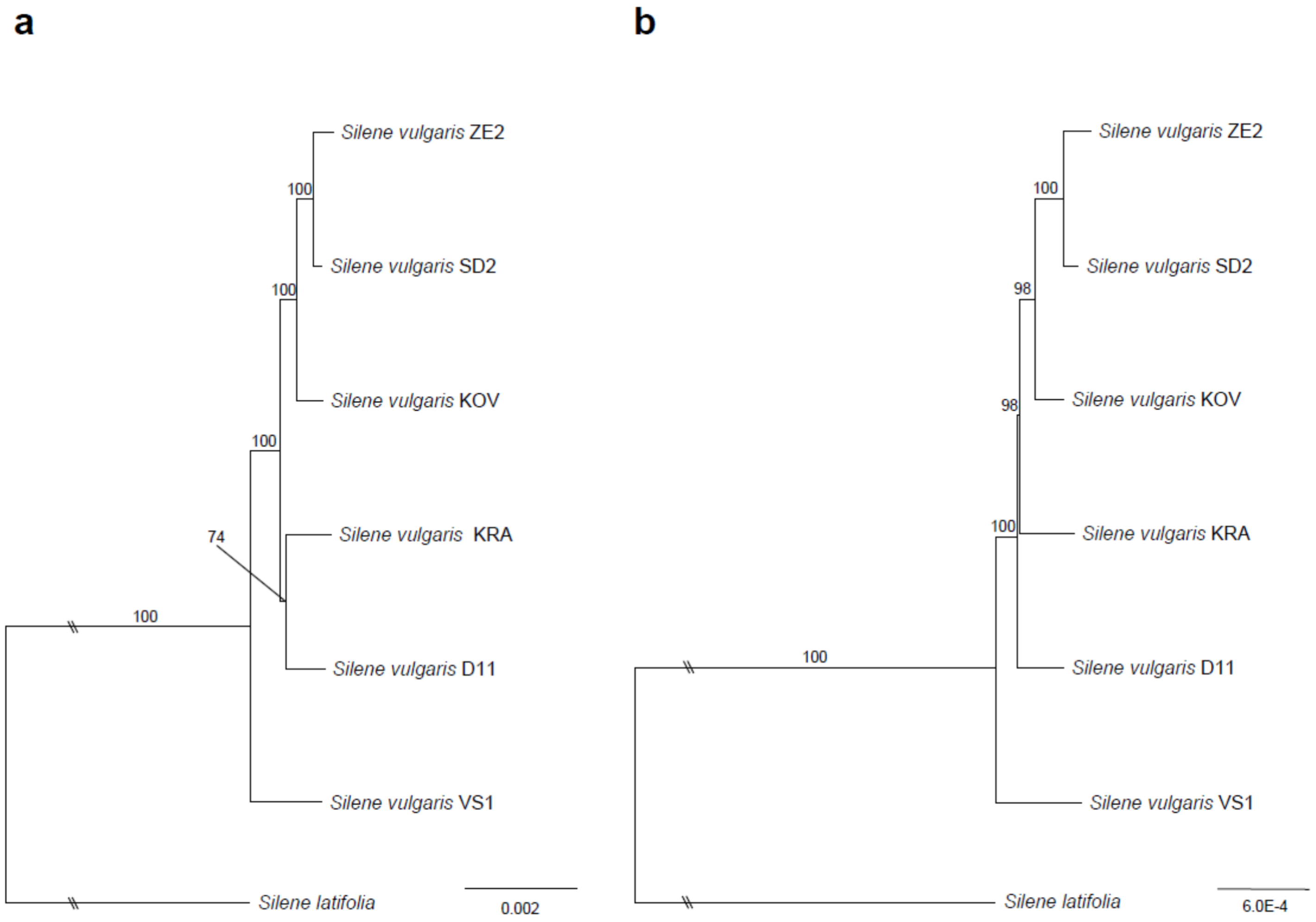

Supplementary Figure S1: Maximum likelihood phylogenetic trees for different palstid haplotypes of *S. vulgaris* **a** based on all sites of the plastid genome except for homopolymer sites larger than five nucleotides; **b** plastid coding regions only. *Silene latifolia* was used as outgroup. Long branches were shorten by 50%, indicated with two diagonal slashes. Indels were coded after Simmon & Ochoterena (2000). The scale bar indicates the number of substitutions per site. Branches with bootstrap support below 60% were collapsed to polytomies. Phylogenetic trees were computed through the CIPRES webportal with RAxML v. 8.2.10 using 1000 bootstraps and the GTRGAMMA model.
