# Supplementary material for: Variation in plastid genomes in the gynodioecious species Silene vulgaris
Source: BMC Plant Biol. 2019 Dec 19;19:568. doi: 10.1186/s12870-019-2193-0 (PMC6921581; doi:10.1186/s12870-019-2193-0)
Supplement: Supplementary file 3 — Additional file 3: Figure S2. Bayesian 50% majority rule phylogenetic trees for different plastid haplotypes of Silene vulgaris based on a. all sites of the plastid genome except homopolymer regions larger than five nucleotides; b. plastid coding regions only; c. all sites of the plastid genome except homopolymer regions larger than five nucleotides with simple indel coding after Simmon & Ochoterena (2000); d. plastid coding regions only with simple indel coding. Silene latifolia was used as outgroup. Long branches were shorten by 50%, indicated with two diagonal slashes. The scale bar indicates the number of substitutions per site. Phylogenetic trees were computed through the CIPRES webportal with MrBayes v. 3.2.6 using 5000 generations [file 12870_2019_2193_MOESM3_ESM.pdf]

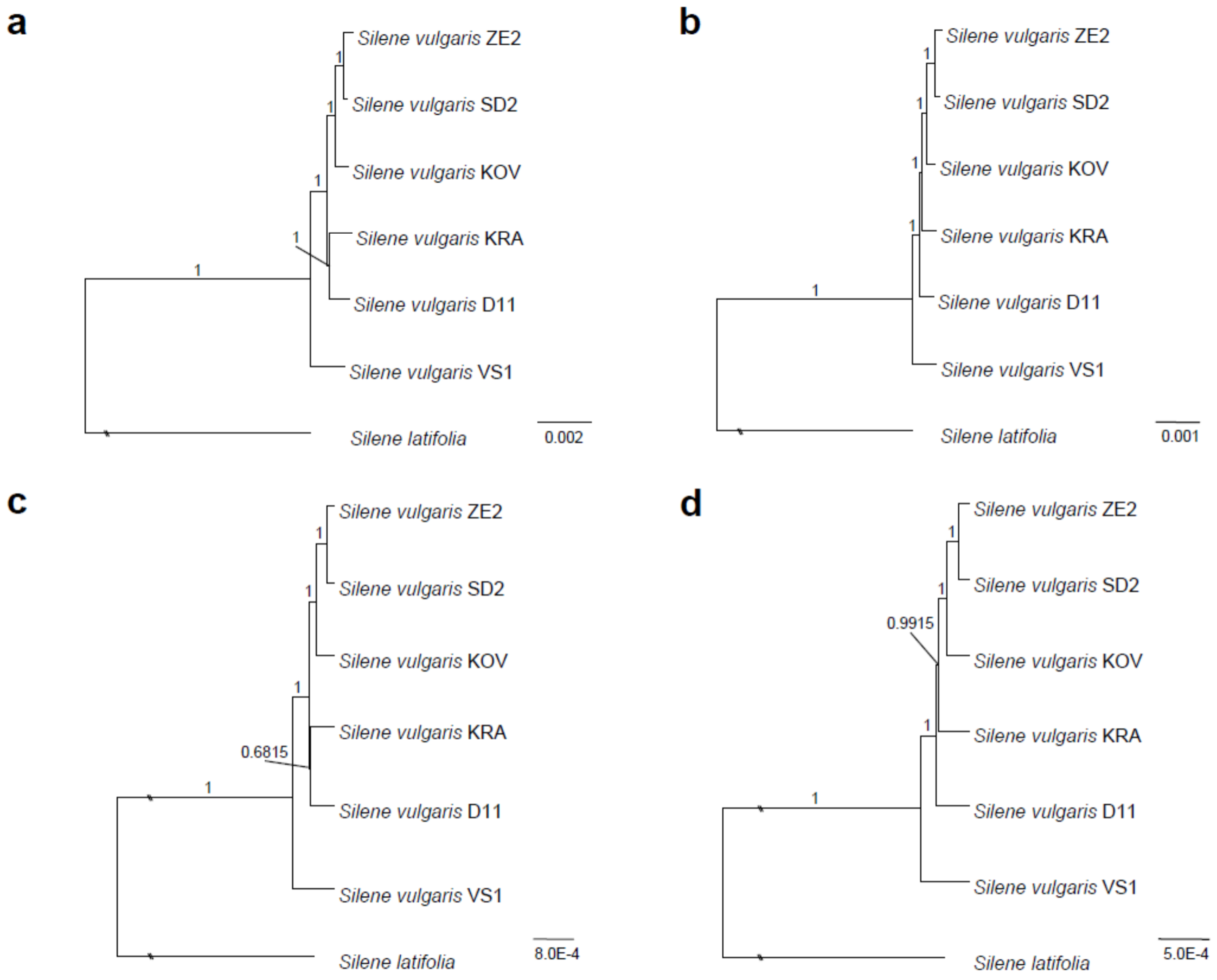

Supplementary Figure S2. Bayesian 50% majority rule phylogenetic trees for different plastid haplotypes of *S. vulgaris* based on **a** all sites of the plastid genome except homopolymer regions larger than five nucleotides; **b** plastid coding regions only; **c** all sites of the plastid genome except homopolymer regions larger than five nucleotides with simple indel coding after Simmon & Ochoterena (2000); **d** plastid coding regions only with simple indel coding.

Silene latifolia was used as outgroup. Long branches were shorten by 50%, indicated with two diagonal slashes. The scale bar indicates the number of substitutions per site. Phylogenetic trees were computed through the CIPRES webportal with MrBayes v. 3.2.6 using 5000 generations.
