# Supplementary material for: Variation in plastid genomes in the gynodioecious species Silene vulgaris
Source: BMC Plant Biol. 2019 Dec 19;19:568. doi: 10.1186/s12870-019-2193-0 (PMC6921581; doi:10.1186/s12870-019-2193-0)
Supplement: Supplementary file 4 — Additional file 4: Figure S3 Heat maps showing the transcript levels of the plastid ndh genes across six individuals of S. vulgaris KRA and KOV. The ndhF gene is the least expressed gene in both haplotypes, the expression of the other genes varies between the two haplotypes [file 12870_2019_2193_MOESM4_ESM.pdf]

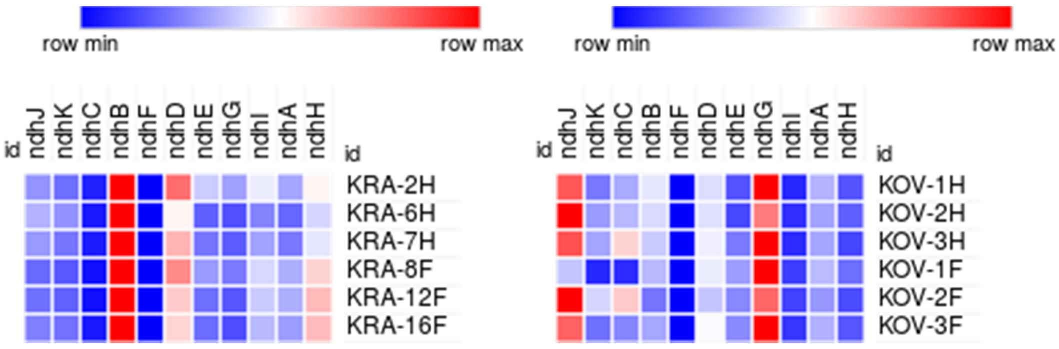


**Supplementary Figure S3.** Heat maps showing the transcript levels of the plastid *ndh* genes across six individuals of *S. vulgaris* KRA and KOV. The *ndhF* gene is the least expressed gene in both haplotypes, the expression of the other genes varies between the two haplotypes.
