# Supplementary material for: Variation in plastid genomes in the gynodioecious species Silene vulgaris
Source: BMC Plant Biol. 2019 Dec 19;19:568. doi: 10.1186/s12870-019-2193-0 (PMC6921581; doi:10.1186/s12870-019-2193-0)
Supplement: Supplementary file 5 — Additional file 5: Figure S4. The comparison of editing rates estimated by the GSNAP and the ChloroSeq pipeline. Mean values (±SD) calculated from six individuals are plotted, a 95% confidence band for the observed data is given in gray within plot [file 12870_2019_2193_MOESM5_ESM.pdf]

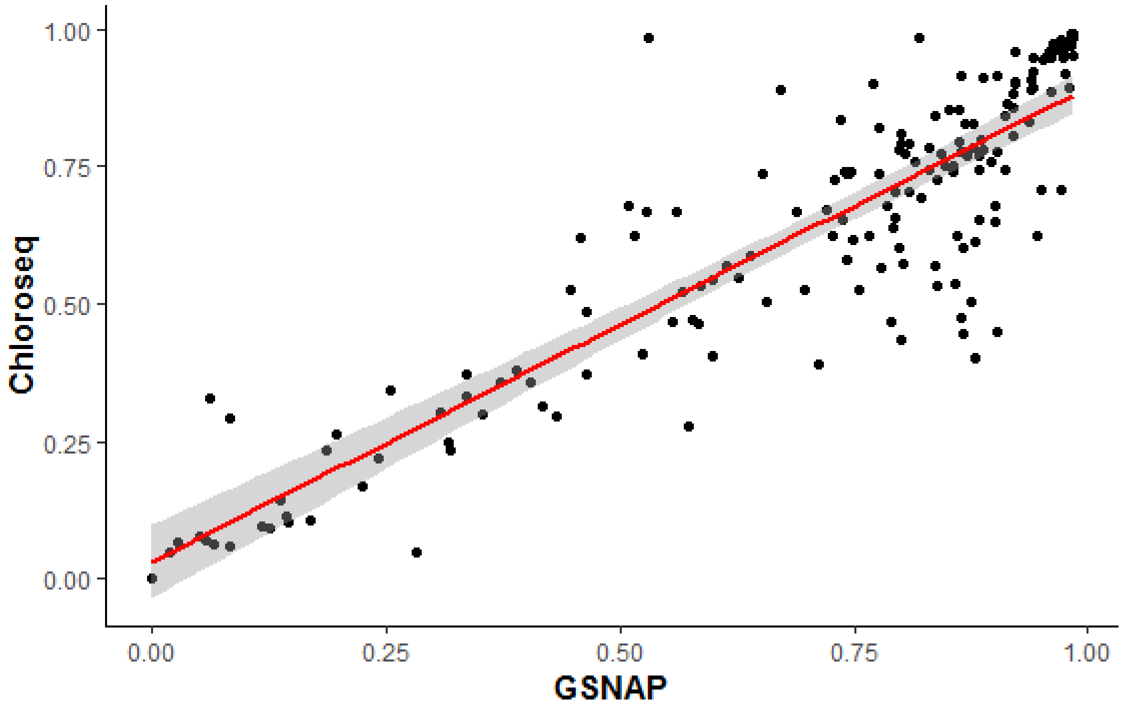


**Supplementary Figure S4.** RNA editing extent in *S. vulgaris* plastids estimated by the GSNAP and ChloroSeq pipelines in the haplotypes KOV and KRA. Mean values (±SD) calculated from six individuals are plotted, a 95% confidence band for the observed data is given in gray within plot.
